# Sex differences in brain activation patterns with mental stress in patients with coronary artery disease

**DOI:** 10.1186/s13293-019-0248-4

**Published:** 2019-07-12

**Authors:** Nicole Kasher, Matthew T. Wittbrodt, Zuhayr S. Alam, Bruno B. Lima, Jonathon A. Nye, Carolina Campanella, Stacy Ladd, Muhammad Hammadah, Amit J. Shah, Paolo Raggi, Arshed A. Quyyumi, Viola Vaccarino, J. Douglas Bremner

**Affiliations:** 1 Department of Epidemiology, Rollins School of Public Health, Emory University, Atlanta, GA USA; 2 Department of Psychiatry and Behavioral Sciences, Emory University School of Medicine, Emory University, Atlanta, GA USA; 3 Department of Medicine (Cardiology), Emory University School of Medicine, Emory University, Atlanta, GA USA; 4 Department of Radiology, Emory University School of Medicine, Emory University, Atlanta, GA USA; 5 Atlanta VA Medical Center, Decatur, GA USA; 6 Mazankowski Alberta Heart Institute and the Department of Medicine, University of Alberta, Edmonton, Alberta Canada

## Abstract

**Background:**

Stress is an important contributor to myocardial ischemia and the progression of coronary artery disease (CAD), and women are more susceptible than men to these effects. Little is known, however, about the neural basis of these sex differences.

**Methods:**

We investigated sex differences in neural correlates of mental stress in a sample of 53 female and 112 male participants (*N* = 165) with CAD, with and without mental stress-induced myocardial ischemia (MSI), during exposure to mental arithmetic tasks and public speaking stress tasks using high-resolution positron emission tomography (HR-PET) and radiolabeled water imaging of the brain.

**Results:**

Women compared to men had significantly greater activation with stress in the right frontal (BA 9, 44), right parietal lobe (Area 3, 6, 40), right posterior cingulate gyrus (BA 31), bilateral cerebellum, and left temporal/fusiform gyrus (BA 37) and greater deactivation in bilateral anterior cingulate gyrus (BA 24, 32), bilateral medial frontal gyrus (BA 6, 8, 9, 10), right parahippocampal gyrus, and right middle temporal gyrus (BA 21). Women with MSI (but not those without MSI) showed significantly greater activation than men in the right posterior cingulate gyrus (BA 31) and greater deactivation in several frontal and temporal lobe areas.

**Conclusion:**

Men and women with CAD show differences in responses to stress in brain limbic areas that regulate emotion, and these functional responses differ by MSI status. Our results suggest that the cingulate gyrus may be involved in sex differences in MSI.

**Electronic supplementary material:**

The online version of this article (10.1186/s13293-019-0248-4) contains supplementary material, which is available to authorized users.

## Introduction

According to estimates from the Global Burden of Disease 2010 Study, mental health-related disorders and cardiovascular disease (CVD) are the leading contributors to morbidity and mortality worldwide [1, 2]. Furthermore, mental health disorders including depression are associated with a greater incidence of coronary artery disease (CAD) and poorer prognosis after myocardial infarction (MI) and are generally more pronounced in women than men [3–10]. Sex differences in brain and physiological responses to stress in CAD patients may represent a mechanism for these differences between men and women in the incidence, prognosis, and pathophysiology of CAD.

Clinical characteristics of CAD and mental illness differ in men and women [11]. Generally, while women younger than 65 years of age are less likely to develop CAD compared to men, once they are diagnosed with CAD, younger women tend to have a greater number of comorbidities and cardiovascular risk factors, longer duration of hospitalization for CAD, and greater risk of mortality within 30 days of hospitalization, even though they have less coronary atherosclerosis [3, 11–20].

A clear distinction between women and men with CAD is that women have a higher burden of stress, depression, and anxiety [21–23]. Furthermore, recent experimental studies [11, 21, 24] have shown that psychological stress may differentially or disproportionately affect women with CAD more than men. Mental stress-induced myocardial ischemia (MSI) is a phenomenon characterized by a demand-perfusion mismatch of the heart during a mental stress challenge [25, 26]. MSI is associated with poorer prognosis and increased mortality among patients with CAD. In a series of studies, we have shown that MSI is more common in women with CAD, especially young women, and that the underlying mechanisms may differ in women and men [21, 24, 25, 27–29]

The neural pathways linking mental stress to CAD have only recently been the subject of investigation. Brain regions involved in emotion and cardiovascular regulation, including the medial prefrontal cortex, insula, and amygdala, have been hypothesized to play a role in MSI [11]. Increased activity in the amygdala has been linked to both exposure to early stress [30–36] and PTSD [30–41] and the development of CVD [42]. We have shown increased rostral anterior cingulate (medial prefrontal cortex) activation with stress in CAD patients with MSI [43]. Prior imaging studies in conjunction with stress have shown sex differences in brain reactivity in healthy adults in brain areas involved in emotion, including the amygdala, hippocampus, and medial prefrontal cortex [44–46]. The neural correlates of stress comparing men and women with CAD, and with and without MSI, however, are not known. The objective of the present study was to investigate sex differences in neural correlates of mental stress and MSI in male and female patients with CAD. We addressed whether there are sex-related differences in the brain’s response to mental stress in patients with CAD, and whether these differences are modified by MSI. We hypothesized that women would show greater activation with stress than men in brain areas involved in modulation of emotion, fear, and peripheral autonomic and stress reactivity, including amygdala, insula, and medial prefrontal cortex (anterior cingulate), and that these differences would be more pronounced in those subjects who develop MSI.

## Methods

### Study design

Participants were 60 female and 126 male patients with known CAD (*N* = 186) that were participants in the larger Mental Stress Ischemia Mechanisms and Prognosis Study (MIPS). Detailed methods for the MIPS cohort were described elsewhere [47]. Briefly, 695 patients between 30 and 80 years of age with confirmed stable coronary artery disease (CAD) were prospectively enrolled between 2011 and 2014 from Emory University Hospital, Grady Memorial Hospital, and the Atlanta VA Medical Center. A clinical diagnosis of CAD was met if participants had angiographic evidence of CAD with at least one major vessel affected, a history of myocardial infarction, coronary bypass surgery or angioplasty, or a positive nuclear scan or exercise test. Patients were excluded from the current study if they had a history of a major psychiatric illness, based on the Structured Clinical Interview for the Diagnostic and Statistical Manual IV (SCID), including schizophrenia, schizoaffective, or bipolar disorder, and a recent history of alcohol or substance abuse or dependence in the past year. Patients were also excluded if they had a history of loss of consciousness exceeding 1 min, meningitis, neurological disorder such as Parkinson’s disease or dementia, chronic oral steroid use or inhaled steroid use greater than 1500 μg/day, or antipsychotic, opiate, or benzodiazepine medication use within the past month. The parent study’s aim, of the which this sample was a sub-group, was to over represent a history of depression so that approximately half of the sample would have depression (either current depressive episode or a Beck Depression Inventory score > 13). For that reason and based on methodological considerations outlined elsewhere [48], patients currently on antidepressant medications were not excluded from the study. Patients who were positive for MSI as part of the main MIPS protocol were also oversampled for the current study, resulting in roughly equivalent rates of MSI, unlike the parent study in which we have previously reported in this same sample higher rates of MSI in women, especially younger (< 50) women [21]. All patients underwent mental stress testing and myocardial perfusion during rest and stress was measured with Tc-99m sestamibi and Single Photon Emission Computed Tomography (SPECT) using a standardized protocol described previously in detail [47]. All study subjects provided informed consent and the study was approved by the Emory University Institutional Review Board.

### Psychometric assessments

The Structured Clinical Interview for DSM IV (SCID) [49] was administered at the baseline visit by trained personnel to establish a depression diagnosis. Sociodemographic characteristics, medical history, and medication use were collected by a research nurse using standard questionnaires, chart reviews, and in-person interviews.

### Mental stress testing

Subjects underwent eight PET brain imaging scans in conjunction with mental stress and control tasks in a single day. Participants were asked to hold beta-adrenergic antagonists and nitrate and calcium channel blockers for a minimum of 12–24 hours prior to mental stress testing. Subjects were scanned twice for each of the four tests (mental arithmetic control, public speaking control, mental arithmetic stress, and public speaking stress). After resting in a quiet room for 30 min, subjects were asked to perform the neutral control tasks and then the stressful tasks, each lasting approximately 2 min, and were scanned during each task. The order of the mental stresses was randomized. For the mental arithmetic control condition, participants were tasked with counting out loud. For the public speaking control condition, subjects discussed a neutral event. For the mental stress arithmetic task, participants were asked to solve a series of increasingly complex math problems under a time constraint and were given negative feedback regarding their performance by a white-coated staff member administering the test [50]. To ensure that all participants experienced similar stress levels independent of personal skill, the difficulty level of arithmetic problems was increased until patients incorrectly answered three consecutive math problems. For the public speaking task, participants were provided two scripted scenarios of stressful interpersonal situations and instructed to develop a speech regarding these events. They were given 2 min to prepare each speech and three min to present it to an audience. Subjects were told that the content and duration of their speeches would later be evaluated.

### Brain imaging during stress

Subjects underwent high-resolution positron emission tomography (HR-PET) brain imaging with the high-resolution research tomograph (HRRT) (CTI, Knoxville, TN), with a spatial resolution of 2 mm [51]. There was a total of eight brain scans, two scans during each of the two control (counting aloud and recalling a neutral event) and two stress (arithmetic and public speaking) conditions. Subjects were injected with 20 mCi of ^15^O water 10 s after the beginning of each task to assess brain function.

### Myocardial perfusion imaging during stress

On a separate day, subjects completed single-photon emission computed tomography (SPECT) cardiac imaging in conjunction with a public speaking task to measure myocardial perfusion at rest and with mental stress, using methods previously described as part of the MIPS protocol [47]. Patients were injected with 10–14 mCi of [Tc-99m] sestamibi at rest, and SPECT images of the heart were acquired 30–45 min later. After resting for 30 min, patients completed the public speaking stress task and were injected with 30–40 mCi of [Tc-99m] sestamibi (depending on body weight) 1 min after beginning the task. Images were acquired 40–60 min later. Cardiac data were analyzed according to a 17-myocardial segments model and scored separately by two experienced readers, blinded to the task condition, and without prior knowledge of the subject’s medical history, on a scale of 0 (normal) to 4 (no perfusion). Disagreements were resolved by consensus. Stress scores were calculated by adding numbers in rest and stress conditions and calculating the difference. Participants with a stress score of 3 or higher were determined to be positive for MSI, while those with a score lower than 3 were negative for MSI.

### Hemodynamic reactivity

Hemodynamic reactivity measures were collected using an automatic oscillometric device. Measurements were recorded at baseline and during each control and mental stress task. The measurements obtained were averaged over the control and stress tasks to obtain mean control and mean mental stress measures. The mean rate pressure product during mental stress and control conditions for each subject was calculated as the product of the mean heart rate and the mean systolic blood pressure during control tasks and during mental stress tasks. Stress reactivity for systolic blood pressure, heart rate, and rate pressure product was calculated as the difference between mean mental stress and mean control measures.

### Data analysis

Differences in demographic and clinical variables between men and women were assessed using two sample *t* tests for continuous variables and Chi-square tests for categorical variables. The likelihood ratio test and twoway analysis of variance (ANOVA) were used to calculate the interaction of sex with MSI for categorical and continuous variables, respectively. Generalized linear modeling (GLM) was used to compare hemodynamic reactivity (heart rate, systolic blood pressure, and rate pressure product) between men and women, before and after adjusting for covariates. Variables sequentially added and adjusted for in the models included age, race, body mass index (BMI), history of myocardial infarction, history of heart failure, and antidepressant and beta-blocker use. Variables were selected for inclusion based on a priori considerations that they might confound the association, and they were retained if their inclusion caused at least a 10% change in the estimate for sex.

HR-PET images of brain activation and deactivation during stress in men and women with and without MSI in hypothesized regions (bilateral amygdala, insula, and anterior cingulate/medial prefrontal cortex) were processed using statistical parametric mapping (SPM8) software, following methods previously described [52, 53]. All scans were realigned to the first image in the scanning session, smoothed, and normalized onto a standard brain template from the Montreal Neurological Institute (MNI). First, an individual contrast map was created to identify areas of activation (stress–rest) or deactivation (rest–stress). For the purposes of this study, all control and mental stress tasks were averaged across type. Contrast maps were then computed across between-subject factors (gender, MSI). A two-layered mask was applied to each gender difference by MSI contrast. First, an exclusive mask was applied based upon significant differences during control tasks (Additional file 1: Table S1). Second, an inclusive mask was applied based on the within-gender significant activations or deactivations (Additional file 1: Tables S2–S5) as a result of mental stress. All brain activations were controlled for African-American race, presence of depression, usage of anti-depressants, diuretics, beta-blockers, and history of heart failure. Areas of significant differences based on gender and task were displayed using mricron (nitrc.org/projects/mricron) with standard stereotactical coordinates [54]. Significance MSI and gender contrast thresholds were set at *p* < 0.005 and 11 contiguous voxels in brain regions to minimize risk of Type 1 and Type 11 errors [55] with the exception being within-gender activation/deactivation maps which were family-wise error corrected given the nature of a single main effect regressor contrast using this analytical pipeline [56]. Areas of significant differences based on sex and task were displayed using SPM8 with standard stereotactical coordinates [54]. Significance thresholds were set at *p* < 0.005 and 11 contiguous voxels in brain regions to minimize risk of Type 1 and Type 11 errors [55].

## Results

Of the 186 individuals enrolled in the study, 7 women and 14 men were excluded due to either poor scan quality or incomplete scans. The final analysis included 53 women and 112 men with a mean (± standard deviation) age of 61.2 ± 7.7 and 62.3 ± 8.7, respectively. The proportion of African-Americans was nearly twofold greater among women than men (Table 1 in the “Appendix” section). Women were also more likely to have had heart failure and major depression in their lifetime and to be treated with antidepressant, beta-blocker, and diuretic medications. All other select demographic, clinical, and lifestyle characteristics were evenly distributed among men and women in the dataset, even after stratifying for MSI status (Table 1 in Appendix).

Men and women did not differ significantly in hemodynamic reactivity to psychosocial stress testing (Table 2 in Appendix). At baseline, women, compared to men, had significantly higher heart rate (mean ± standard deviation, 67 ± 10 vs 63 ± 10 bpm, *p* = 0.01) and rate pressure product (9359 ± 1951 vs 8456 ± 1661, *p* = 0.002). During mental stress, women displayed greater average heart rate compared to men (78 ± 13 vs 74 ± 13 bpm, *p* = 0.04). However, there were no statistically significant sex differences in either systolic blood pressure, heart rate or rate pressure product reactivity to mental stress in both unadjusted and adjusted models.

Women had greater baseline activity during the neutral tasks (Additional file 1: Table S1) in the occipital lobe, temporal lobe, parietal lobe, and cerebellum. To account for these differences, only areas outside of the baseline differences were considered to be altered as a result of mental stress. Across the entire sample, men and women showed different neural activation and deactivation in response to mental stress, compared to control conditions. Compared to men, women showed greater activation in the left temporal/fusiform gyrus (BA 37), right parietal lobe (BA 3, 6, 40), right frontal lobe (BA 9, 44), right posterior cingulate gyrus (BA 31), and bilateral cerebellum during mental stress compared to control tasks (Table 3 in Appendix). However, women had greater deactivation than men to mental stress testing in multiple corticolimbic and related structures, including the bilateral anterior cingulate gyrus (BA 24, 32), bilateral medial frontal gyrus (BA 6, 8, 9, 10), right parahippocampal gyrus, and right middle temporal gyrus (BA 21; Table 3 in Appendix).

**Fig. 1:**
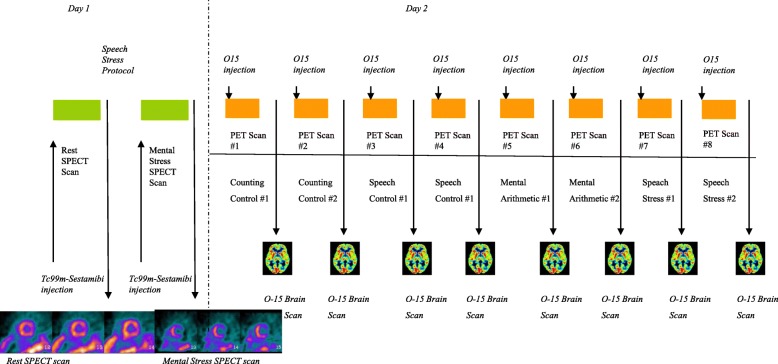
Study diagram for days 1 and 2 of the protocol. On day 1, subjects get an injection of [Tc99m] sestamibi followed by SPECT imaging of the heart at rest. Three hours later they undergo speech mental stress followed by injection of [Tc99m] sestamibi and SPECT imaging of the heart with mental stress. On the second day, subjects undergo HR-PET imaging of the brain with stress and control conditions. Subjects undergo eight HR-PET scans after injection of 20 mCi O-15 water, 2 with counting control, 2 with speech control, 2 with arithmetic mental stress, and 2 with speech mental stress

**Fig. 2:**
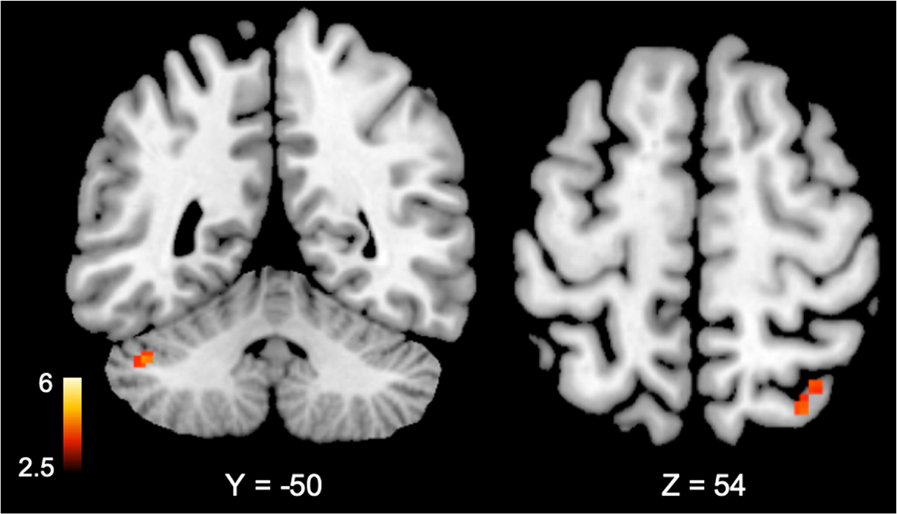
Brain areas with greater (*p* < 0.005) cerebral blood flow increases (activation) during mental stress compared to control tasks in women (*n* = 44) versus men (*n* = 77) with coronary artery disease but no mental stress induced myocardial ischemia using [^15^O]H_2_O positron emission tomography. Values below brain denote Talairach coordinates. Color bars indicate *Z* values of activation or deactivation

**Fig. 3:**
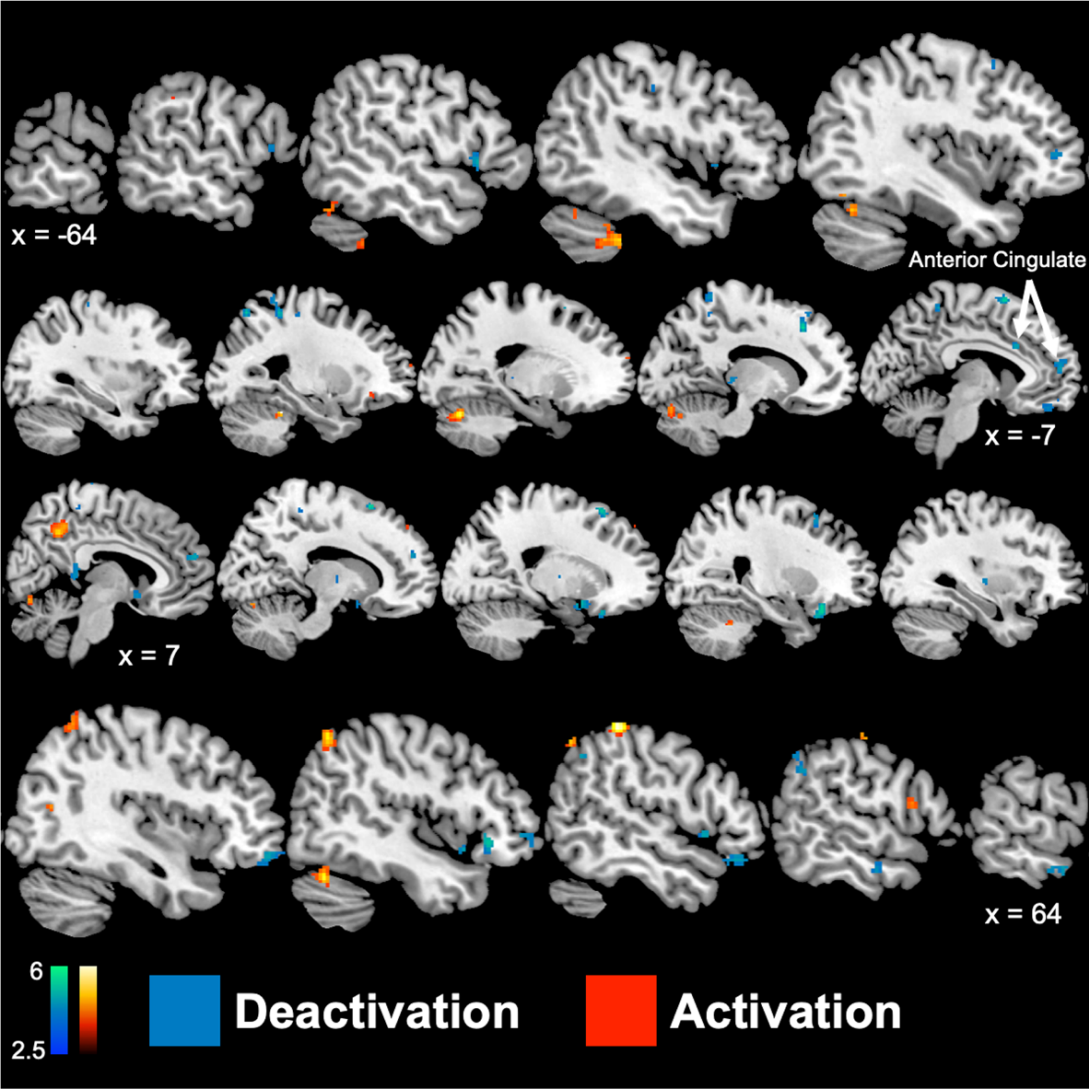
Sagittal brain slices representing greater (*p* < 0.005) cerebral blood flow increases (activation; red) and decreases (deactivation; blue) during mental stress compared to control tasks in women (*n* = 9) versus men (*n* = 35) with coronary artery disease and mental stress induced myocardial ischemia using [^15^O]H_2_O positron emission tomography. Values below brain denote Talairach x-coordinates, where positive and negative values correspond to the right and left hemisphere, respectively. Color bars indicate *Z* values of activation or deactivation

Sex differences in neural reactivity to mental stress also differed by MSI status. Among participants without MSI, sex differences during stress compared to control were observed in the left cerebellum and right superior parietal lobe (Table 4 in Appendix, Fig. 1). In contrast, women with MSI showed greater activation than men with MSI with stress in many brain areas including the right posterior cingulate gyrus (BA 31), right parietal lobe (BA 3, 7, 40), bilateral frontal lobe (BA 6, 8, 9, 10, 11, 44), left temporal lobe (BA 39), and bilateral posterior cerebellum (Table 5 in Appendix, Figs. 2 and 3). Furthermore, women with MSI also had greater deactivation with stress, relative to men with MSI, in the right middle temporal gyrus (BA 21), bilateral superior frontal gyrus (BA 6, 7, 8), bilateral middle frontal gyrus (BA 6, 8, 11), bilateral medial frontal gyrus (BA 6, 9, 10), and the bilateral inferior frontal gyrus (BA 45, 47).

## Discussion

This study showed unique patterns of brain response to stress in men and women with mental stress-induced myocardial ischemia (MSI) compared to non-MSI. Women without MSI had focal but scant increases in activation/deactivation compared to men without MSI. Women with MSI, however, showed widespread changes in brain activity compared to men with MSI including activation of the anterior cingulate, right parietal lobe, and bilateral frontal lobe with stress, and greater deactivations in temporal cortex, amygdala, and superior and inferior, medial, and middle frontal gyrus. These differences were seen in spite of the fact that men and women showed similar cardiovascular reactivity to stress.

The anterior cingulate plays a pivotal role in both the regulation of emotion and peripheral autonomic and cardiovascular responses to stress [57, 58]. In addition to activating peripheral cardiovascular responses that could lead to MSI, this region is involved in the inhibition of fear responses in the amygdala and the extinction of fear [59–62]. Previous studies have shown that chronic stress alters functional reactivity of the cingulate cortex and other subsets of the limbic system to stress [63, 64]. Studies in patients with stress-related psychiatric disorders including PTSD and depression (that have also been linked to cardiovascular disease) [65, 66] found decreases in stress-responsive activation in this area as well as smaller volume [67–69]. In the current study, mental stress was associated with significantly greater deactivation of the left anterior cingulate cortex in women with CAD compared to men with CAD. Greater anterior cingulate deactivations were only observed in those with MSI, as women with CAD and MSI had bilateral deactivations within the anterior cingulate compared to men with CAD.. These results suggest that dysregulation of the anterior cingulate gyrus could be involved in pathways linking mental stress to myocardial ischemia and cardiovascular-related death [70]. The results further suggest that there are differences between men and women, both in those with and without MSI. These findings may help explain why women are at increased risk compared to men for both stress-related psychiatric disorders and MSI.

Unique patterns of brain activation in women versus men with MSI could translate into differences in peripheral stress-responsive neurohormonal and cardiovascular systems. The central autonomic network contributes to the regulation of the autonomic nervous system and has sympathetic and parasympathetic system network subdivisions [71]. In the current study, stress resulted in greater deactivation in brain regions associated with sympathetic regulation (posterior and mid cingulate cortex) as well as greater activation in brain areas tied to parasympathetic regulation (cerebellum) [72, 73] in women with CAD than men with CAD. This relationship appears to occur only in those with MSI. Furthermore, the default mode network, brain regions engaged while performing passive tasks, were also more deactivated in women than men with CAD. Brain areas of the default mode network include the lateral temporal cortex and dorsal medial prefrontal cortex and are involved in functions such as introspection and exploration during low attention-requiring tasks [74]. Our results suggest that women may have been more task-focused and less engaged in internal meditations than men in response to heightened psychosocial stress, and that mechanisms of autonomic regulation in response to stress may differ in men and women.

Men and women had many similar increases in activation and deactivation to psychosocial stress in brain regions that contribute to the execution of emotionally and cognitively stressful tasks. This included areas implicated in mental arithmetic, phonological language processing, visual processing, spatial awareness, working memory, and emotion regulation (middle frontal gyrus, superior frontal gyrus, inferior parietal lobe, supramarginal gyrus, and cingulate gyrus) [75–81]. Women with CAD, however, showed greater activation in response to mental stress in a brain region implicated in language processing (middle temporal gyrus) [78] when compared to men with CAD. Interestingly, mental stress resulted in a greater decrease in blood flow in women than men in brain regions that contributed to the regulation of language and literacy, vision and eye movement, and social cognition (right middle temporal gyrus, BA 21, medial frontal gyrus, BA 8 and 9) [82, 83] and most notably, anterior cingulate gyrus.

Our study provides a foundation for the identification of neural biomarkers of mental stress in men and women with CAD. Our study, however, had limitations worth noting. Participants may have exerted themselves differently during performance of stress or control tasks and this may be a confounder. Men and women in this study differed in several variables, including race and antidepressant usage. These variables, however, may be independently associated with MSI. For instance, women have twice the rates of stress-related psychiatric disorders than men [84], and African-American women with CAD are particularly at risk [85], which may explain higher rates of antidepressant usage as well as MSI. Additionally, we have found that MSI is most common in women, and in particular younger women [24, 28, 29], with a predilection for African-American women, a finding from the parent study of the current cohort [21]. These may represent, therefore, co-variables with sex-based differences in effects of stress on the brain. The findings persisted, however, after controlling for these variables. Another limitation of this study is that the only patients with CAD were included, therefore our results are only generalizable to patient populations with CAD. The findings, however, offer suggestive evidence that differences between men and women in how brain areas modulating emotion, notably the anterior cingulate, respond to stress, may offer clues about differences in stress-induced myocardial ischemia.

## Perspectives and significance

The results of this study indicate that neural correlates of mental stress differ between men and women with CAD, with greater deactivation in women in multiple regions including the anterior cingulate gyrus, a brain region involved in emotional and autonomic regulation, compared to men. These findings related to the anterior cingulate carry over in the comparison between men and women with stress-induced myocardial ischemia. The findings suggest a possible mechanism for important differences between men and women in stress-induced myocardial ischemia, notably that women, especially younger women, may be more susceptible to this phenomenon. The clinical relevance of this is that stress-induced myocardial ischemia may require different treatment approaches than exercise-induced ischemia, such as relaxation training, meditation, biofeedback, or other behavioral approaches. Furthermore, the worse prognosis of these patients, especially for younger women with stress-induced myocardial ischemia than for men and/or exercise-induced ischemia, illustrates the importance of focused approaches to these patients to ensure optimal outcomes.

### Additional file

Additional file 1: Additional tables. (DOCX 59 kb)

## Data Availability

Data availability inquiries can be made to the senior author.

## Appendix

**Table 1:** Demographic and clinical characteristics of study participants by sex (*N* = 165)

	Women	Men	*P*
	(*n* = 53)	(*n* = 112)	
Demography			
Age, y ± SD	61.2 ± 7.7	62.3 ± 8.7	0.42
African-American, %	29 (55)	32 (29)	*0.001*
Education (high school)	12 (23)	33 (29)	0.36
Clinical Characteristics and Lifestyle Factors			
BMI, ± SD	31.2 ± 7.4	29.5 ± 5.2	0.09
Current major depressive episode, SCID, %	17 (32)	24 (22)	0.16
Lifetime major depression, SCID, %	31 (58)	37 (33)	*0.002*
Abnormal angiogram	37 (76)	66 (60)	0.06
Abnormal nuclear test	7 (14)	14 (13)	0.79
Abnormal exercise test, %	9 (18)	13 (12)	0.27
Diabetes, %	19 (36)	38 (34)	0.81
Dyslipidemia, %	40 (75)	96 (86)	0.11
Heart Failure, %	14 (26)	12 (11)	*0.01*
Hypertension, %	40 (75)	85 (76)	0.95
MI History, %	16 (30)	47 (42)	0.15
MSI, %	9 (17)	35 (31)	*0.05*
Smoking History			
Current Smoker, %	7 (14)	16 (15)	0.31
Former Smoker, %	21 (43)	60 (54)	
Never Smoker, %	21 (38)	34 (31)	
Medication Use			
ACE Inhibitors, %	21 (40)	52 (47)	0.38
Antidepressants, %	26 (50)	29 (26)	*0.003*
Aspirin, %	45 (85)	96 (86)	0.78
Beta Blockers, %	45 (85)	77 (69)	*0.03*
Diuretics, %	24 (45)	26 (23)	*0.004*
Statins, %	41 (84)	99 (91)	0.19
Vasodilators, %	5 (9)	12 (11)	0.79

*Abbreviations*: *SPECT* single photon emission computed tomography, *MSI* mental stress ischemia, *BMI* body mass index, *SCID* Structured Clinical Interview for DSM IV, *MI* myocardial infarction

**Table 2:** Sex differences in hemodynamic reactivity parameters in response to mental stress, unadjusted (*N* = 165)

	Women (*n* = 53)	Men (112)	*P* value
	Mean ± SD	Mean ± SD	
Systolic blood pressure (mmHg)			
Control	140 ± 22	135 ± 15	0.07
Stress	162 ± 27	161 ± 21	0.91
Systolic blood pressure reactivity	22 ± 17	27 ± 16	0.07
Heart rate (bmp)			
Control	67 ± 10	63 ± 10	*0.01*
Stress	78 ± 13	74 ± 13	*0.04*
Heart rate reactivity	11 ± 9	11 ± 7	0.81
Rate pressure product (mmHg*bmp)			
Control	9359 ± 1951	8456 ± 1661	*0.002*
Stress	12,676 ± 3065	11,874 ± 2667	0.08
Rate pressure product reactivity	3315 ± 2155	3391 ± 2112	0.82

*SD* standard deviation

^*^Statistical tests: Student t test or Wilcoxon–Mann–Whitney U test, when appropriate

**Table 3:** Brain regions with significantly (one tailed *Z* > 2.75, *p* < 0.005) greater mental stress-induced activation or deactivation in women (*n* = 53) relative to men (*n* = 112) with coronary artery disease as measured with [^15^O]H_2_O positron emission tomography

Voxel number	Brain regions	Brodmann’s area	Talairach			*Z* score
			*X*	*Y*	*Z*	
Greater stress activation in women than men						
22	L cerebellum		− 26	− 40	− 18	5.26
86	L cerebellum		− 18	− 69	− 18	4.85
	L cerebellum		− 16	− 76	− 13	3.61
42	R parietal lobe, postcentral gyrus	40	51	− 32	50	4.75
26	R cerebellum		46	− 63	− 19	4.25
36	R cerebellum		10	− 72	− 10	4.25
11	R parietal lobe, postcentral gyrus	3	53	− 9	49	4.13
	R frontal lobe, precentral gyrus	6	48	− 7	54	2.82
96	L cerebellum		− 44	− 48	− 26	3.87
	L cerebellum		− 48	− 40	− 32	3.52
66	R posterior cingulate	31	8	− 43	39	3.74
	R parietal lobe, precuneus	7	4	− 51	38	3.54
12	L occipital lobe, fusiform gyrus	19	− 42	− 65	− 9	3.73
14	R frontal lobe, precentral gyrus	6	59	− 10	42	3.72
22	R cerebellum		24	− 50	− 23	3.56
19	R frontal lobe, superior gyrus	9	42	37	32	3.55
	R frontal lobe, middle gyrus	9	42	46	26	2.66
33	R parietal lobe, inferior lobule	40	48	− 58	48	3.53
68	R parietal lobe, superior lobule	7	30	− 59	53	3.51
	R parietal lobe, inferior lobule	40	38	− 52	53	3.38
19	R cerebellum		22	− 75	− 15	3.47
32	L temporal lobe, fusiform gyrus	37	− 50	− 61	− 17	3.44
12	R frontal lobe, precentral gyrus	44	59	10	13	3.28
Greater stress deactivation in women than men						
23	L anterior cingulate	24	− 4	23	24	5.02
51	R frontal lobe, superior gyrus	6	14	22	51	5.00
95	R frontal lobe, inferior gyrus	47	24	22	− 20	4.67
	R frontal lobe, orbital gyrus	47	22	30	− 23	3.07
64	L frontal lobe, medial gyrus	6	− 14	29	36	4.63
	L frontal lobe, superior gyrus	8	− 10	32	47	3.50
86	R frontal lobe, inferior gyrus	47	44	27	− 8	4.53
	R frontal lobe, inferior gyrus	47	50	28	− 13	4.04
46	L frontal lobe, superior gyrus	6	− 6	14	55	4.46
	L frontal lobe, superior gyrus	6	0	11	58	3.25
28	L parietal lobe, supramarginal gyrus	40	− 63	− 43	27	4.39
	L temporal lobe, supramarginal gyrus	40	− 63	− 51	23	3.35
49	L frontal lobe, inferior gyrus		− 53	18	1	4.06
17	L parietal lobe, superior lobule	7	− 26	− 64	50	4.00
32	R temporal lobe, middle gyrus	21	69	− 45	− 8	3.99
37	L parietal lobe, precuneus	7	− 14	− 44	59	3.84
26	R frontal lobe, medial gyrus	9	22	38	23	3.84
34	L frontal lobe, medial gyrus	10	− 6	55	7	3.81
34	R temporal lobe, middle gyrus	21	61	− 3	− 17	3.74
11	R anterior cingulate	32	2	27	34	3.73
26	R frontal lobe, subcallosal gyrus	34	18	7	− 12	3.68
13	L frontal lobe, superior gyrus	8	0	28	49	3.67
36	R frontal lobe, medial gyrus	10	10	55	17	3.55
	R frontal lobe, medial gyrus	9	2	46	18	2.82
51	R frontal lobe, middle gyrus	11	42	42	− 19	3.54
	R frontal lobe, middle gyrus	11	38	52	− 14	3.42
31	L parietal lobe, postcentral gyrus	5	− 30	− 44	58	3.53
14	L frontal lobe, middle gyrus	6	− 38	12	46	3.53
14	L frontal lobe, inferior gyrus	45	− 59	18	6	3.50
18	L frontal lobe, precentral gyrus	4	− 44	− 18	33	3.46
13	L frontal lobe, superior gyrus	8	− 22	16	48	3.44
24	R frontal lobe, middle gyrus	8	30	16	41	3.33
29	R parietal lobe, supramarginal gyrus	40	57	− 47	31	3.30
	R parietal lobe, inferior lobule	40	51	− 51	38	3.15
11	L parietal lobe, precuneus	7	− 10	− 40	47	3.27
12	R frontal lobe, medial gyrus	10	2	54	− 9	3.15
15	L parietal lobe, postcentral gyrus	3	− 28	− 23	46	3.13
11	L frontal lobe, inferior gyrus		− 40	43	3	2.99
17	L frontal lobe, middle gyrus	11	− 38	50	− 14	2.95
13	R parahippocampal gyrus	30	8	− 39	7	2.77

**Table 4:** Brain regions with significantly (one tailed *Z* > 2.75, *p* < 0.005) greater mental stress-induced activation or deactivation, as measured with [^15^O]H_2_O positron emission tomography, in women (*n* = 44) relative to men (*n* = 77) participants with CAD but not mental stress-induced myocardial ischemia

Voxel number	Brain region	Brodmann’s area	Talairach			*Z* score
			*X*	*Y*	*Z*	
Stress activation in women > men						
13	L cerebellum		− 44	− 50	− 34	3.56
17	R parietal lobe, superior lobule	7	30	− 63	53	3.19
	R parietal lobe, superior lobule	7	34	− 56	55	3.12

**Table 5:** Brain regions with significantly (one tailed *Z* > 2.75, *p* < 0.005) greater mental stress-induced activation or deactivation, as measured with [^15^O]H_2_O positron emission tomography, in women (*n* = 9) relative to men (*n* = 35) with CAD and mental stress-induced myocardial ischemia

Voxel number	Brain region	Brodmann’s area	Talairach			*Z* score
			*X*	*Y*	*Z*	
Stress activation in women > men						
24	L cerebellum		− 26	− 40	− 18	5.70
54	R parietal lobe, postcentral gyrus	40	51	− 32	50	5.44
112	L cerebellum		− 18	− 69	− 18	5.05
	L cerebellum		− 14	− 76	− 13	3.54
67	R parietal lobe, inferior lobule	40	48	− 58	46	4.97
35	R cerebellum		46	− 63	− 19	4.53
33	R cerebellum		10	− 72	− 10	4.38
112	R posterior cingulate	31	8	− 43	41	4.35
	R parietal lobe, precuneus	7	4	− 51	38	4.18
12	L occipital lobe, fusiform gyrus	19	− 42	− 65	− 9	4.13
110	L cerebellum		− 46	− 38	− 32	4.07
	L cerebellum		− 44	− 48	− 25	3.33
	L cerebellum		− 53	− 44	− 35	3.14
47	L cerebellum		− 42	− 61	− 17	4.02
36	R frontal lobe, inferior gyrus	44	59	12	13	3.91
14	R temporal lobe, middle gyrus	39	40	− 65	15	3.81
15	R parietal lobe, postcentral gyrus	3	59	− 13	44	3.77
20	R frontal lobe, superior gyrus	9	42	37	32	3.66
15	L frontal lobe, superior gyrus	10	− 22	63	12	3.54
19	R cerebellum		24	− 50	− 24	3.54
41	R parietal lobe, inferior lobule	40	38	− 52	53	3.48
15	L frontal lobe, middle gyrus	11	− 24	32	− 12	3.37
13	R frontal lobe, superior gyrus	8	14	48	36	3.33
12	L parietal lobe, inferior lobule	40	− 61	− 33	33	3.26
14	R cerebellum		22	− 75	− 15	3.18
14	R frontal lobe, superior gyrus	10	42	48	23	2.89
Stress deactivation in women > men						
129	R frontal lobe, inferior gyrus	47	24	22	− 20	5.66
	R frontal lobe, orbital gyrus	47	22	30	− 23	4.01
51	R frontal lobe, superior gyrus	6	14	22	51	5.47
37	L frontal lobe, medial gyrus	6	− 14	29	36	4.95
88	R frontal lobe, inferior gyrus	47	44	27	− 6	4.94
	R frontal lobe, inferior gyrus	47	50	34	− 15	3.80
33	L parietal lobe, supramarginal gyrus	40	− 63	− 43	27	4.91
52	L frontal lobe, superior gyrus	6	− 4	12	55	4.56
24	L anterior cingulate	24	− 4	23	24	4.54
17	L parietal lobe, superior lobule	7	− 26	− 64	50	4.41
42	R temporal lobe, middle gyrus	21	69	− 45	− 8	4.25
	R temporal lobe, inferior gyrus	20	67	− 47	− 15	2.82
69	L parietal lobe postcentral gyrus	40	− 26	− 38	49	4.20
	L parietal lobe, postcentral gyrus	5	− 30	− 44	58	3.75
30	L frontal lobe, precentral gyrus	44	− 63	12	9	4.17
	L frontal lobe, inferior gyrus	45	− 59	18	6	3.94
53	R temporal lobe, inferior gyrus	21	61	− 9	− 16	4.16
54	R frontal lobe, subcallosal gyrus	34	16	7	− 14	4.15
24	R frontal lobe, medial gyrus	9	22	38	23	4.04
14	L frontal lobe, superior gyrus	8	0	28	49	3.98
53	L frontal lobe, inferior gyrus		− 53	18	1	3.97
	L frontal lobe, inferior gyrus	47	− 46	14	− 1	3.15
46	R frontal lobe, medial gyrus	10	10	53	16	3.97
26	R frontal lobe, middle gyrus	8	28	16	43	3.93
	R frontal lobe, middle gyrus	8	24	22	46	3.24
15	L frontal lobe, superior gyrus	8	− 22	16	48	3.83
86	R frontal lobe, middle gyrus	10	44	50	− 7	3.80
	R frontal lobe, middle gyrus	11	40	46	− 17	3.63
32	L thalamus		− 16	− 27	3	3.78
32	R parietal lobe, supramarginal gyrus	40	59	− 47	29	3.73
	R parietal lobe, inferior lobule	40	51	− 52	38	3.60
11	R limbic lobe, cingulate gyrus	32	2	27	34	3.72
30	R temporal lobe, superior gyrus	22	51	12	− 2	3.70
	R temporal lobe, superior gyrus	38	46	13	− 9	3.62
35	L frontal lobe, paracentral lobule	5	− 12	− 42	59	3.69
31	L frontal lobe, medial gyrus	10	− 6	55	7	3.64
20	R lentiform nucleus		16	− 7	5	3.61
34	L frontal lobe, paracentral lobule	5	− 6	− 38	52	3.61
	L parietal lobe, precuneus	7	− 12	− 42	47	3.53
51	R posterior cingulate	29	4	− 36	13	3.53
12	L frontal lobe, middle gyrus	6	− 38	12	46	3.41
18	R anterior cingulate	25	4	9	− 7	3.39
14	L frontal lobe, inferior gyrus	10	− 42	45	3	3.39
20	L frontal lobe, precentral gyrus	4	− 44	− 18	36	3.39
12	L frontal lobe, precentral gyrus	4	− 36	− 24	55	3.38
22	L parietal lobe, postcentral gyrus	3	− 30	− 27	42	3.26
22	R frontal lobe, medial gyrus	6	2	− 22	63	3.25
27	L frontal lobe, medial gyrus	8	− 12	31	45	3.22
	L anterior cingulate	32	− 10	22	44	3.22
13	R frontal lobe, medial gyrus	11	2	54	− 11	3.20
11	R lentiform nucleus		32	− 13	2	3.19
17	R frontal lobe, paracentral Lobule	5	8	− 34	54	3.15
	R frontal lobe, paracentral lobule	5	14	− 34	49	2.93
21	L frontal lobe, orbital gyrus	11	− 6	44	− 19	3.10

## Abbreviations

ANOVA: Analysis of variance
BA: Brodmann’s area
BMI: Body mass index
CAD: Coronary artery disease
CVD: Cardiovascular disease
DSM-5: Diagnostic and Statistical Manual – 5
FDA: Food and Drug Administration
GLM: general linear model
HR-PET: High-resolution positron emission tomography
MI: Myocardial infarction
MNI: Montreal Neurological Institute
MRI: Magnetic resonance imaging
MSI: Mental stress ischemia
PET: Positron emission tomography
SCID: Structured Clinical Interview for DSM-5
SPECT: Single-photon emission computed tomography
SPM8: Statistical parametric mapping - 8
