# Supplementary material for: Sex differences in brain activation patterns with mental stress in patients with coronary artery disease
Source: Biol Sex Differ. 2019 Jul 12;10:35. doi: 10.1186/s13293-019-0248-4 (PMC6626382; doi:10.1186/s13293-019-0248-4)
Supplement: Supplementary file 1 — Additional tables. (DOCX 59 kb) [file 13293_2019_248_MOESM1_ESM.docx]

**Additional file 1: Additional Tables**

**Table S1:** Brain areas with significantly (p < 0.005) greater activity during control tasks compared to men as measured with [^15^O]H_2_O positron emission tomography. Cluster peaks are identified using Talairach Atlas coordinates.

|  |  | |  | **Talairach** | | |  |  |
| --- | --- | --- | --- | --- | --- | --- | --- | --- |
| **Voxel Number** | **Brain Region** | | **Brodmann Area** | **X** | **Y** | **Z** | **Z Score** |  |
| 279 | L Occipital Lobe, Middle Gyrus | | 18 | -34 | -93 | 11 | 4.56 |  |
|  | L Occipital Lobe, Superior Gyrus | | 19 | -34 | -88 | 23 | 4.43 |  |
|  | L Occipital Lobe, Middle Gyrus | | 19 | -42 | -89 | 6 | 4.29 |  |
| 181 | L Temporal Lobe, Middle Gyrus | | 21 | -63 | 1 | -17 | 4.35 |  |
|  | L Temporal Lobe, Inferior Gyrus | | 20 | -63 | -5 | -22 | 3.78 |  |
|  | L Temporal Lobe, Middle Gyrus | | 21 | -65 | -7 | -15 | 3.39 |  |
| 143 | R Temporal Lobe, Superior Gyrus | | 38 | 59 | 14 | -26 | 4.14 |  |
|  | R Temporal Lobe, Inferior Gyrus | | 21 | 67 | -7 | -20 | 3.12 |  |
|  | R Temporal Lobe, Middle Gyrus | | 21 | 65 | 5 | -19 | 3.10 |  |
| 2461 | R Parahippocampal Gyrus | | 35 | 20 | -35 | -5 | 4.03 |  |
|  | L Cerebellum | |  | -18 | -47 | -9 | 3.97 |  |
|  | R Cerebellum | |  | 6 | -37 | -8 | 3.91 |  |
| 122 | L Frontal Lobe, Middle Gyrus | | 47 | -48 | 48 | -7 | 4.00 |  |
| 39 | R Occipital Lobe, Superior Gyrus | | 19 | 34 | -86 | 33 | 3.72 |  |
|  | R Occipital Lobe, Superior Gyrus | | 19 | 42 | -82 | 26 | 3.35 |  |
| 92 | R Cerebellum | |  | 53 | -73 | -25 | 3.70 |  |
| 59 | L Cerebellum | |  | -22 | -33 | -37 | 3.53 |  |
|  | L Cerebellum | |  | -30 | -35 | -39 | 3.16 |  |
| 131 | R Occipital Lobe, Lingual Gyrus | | 19 | 30 | -68 | -3 | 3.52 |  |
| 70 | L Parietal Lobe, Precuneus | | 7 | -2 | -77 | 44 | 3.49 |  |
|  | L Parietal Lobe, Precuneus | | 7 | -8 | -77 | 49 | 3.00 |  |
| 100 | L Cerebellum | |  | -46 | -79 | -26 | 3.44 |  |
| 109 | R Cerebellum | |  | 6 | -91 | -24 | 3.40 |  |
|  | R Cerebellum | |  | 24 | -91 | -26 | 3.13 |  |
|  | L Cerebellum | |  | -8 | -91 | -24 | 2.87 |  |
| 203 | L Occipital Lobe, Lingual Gyrus | |  | -20 | -74 | 0 | 3.39 |  |
| 94 | R Temporal Lobe, Inferior Gyrus | | 37 | 48 | -54 | -1 | 3.37 |  |
| 79 | R Cerebellum | |  | 32 | -37 | -38 | 3.31 |  |
|  | R Cerebellum | |  | 24 | -33 | -35 | 2.93 |  |
| 11 | R Temporal Lobe, Middle Gyrus | | 39 | 53 | -71 | 22 | 3.21 |  |
| 272 | L Caudate | |  | -8 | 12 | 4 | 3.18 |  |
|  | L Caudate | |  | -10 | 4 | 10 | 3.13 |  |
| 14 | L Temporal Lobe, Middle Gyrus | | 37 | -38 | -60 | 1 | 3.16 |  |
| 24 | L Temporal Lobe, Inferior Gyrus | | 20 | -65 | -30 | -17 | 3.08 |  |
|  | L Temporal Lobe, Middle Gyrus | | 21 | -69 | -26 | -12 | 2.66 |  |
| 18 | L Occipital Lobe, Lingual Gyrus | | 19 | -32 | -70 | 2 | 3.06 |  |
| 17 | L Temporal Lobe, Inferior Gyrus | | 20 | -57 | -55 | -12 | 2.78 |  |
|  | | | | | | | | |
|  | | | | | | | | |
|  | |  |  |  |  |  |  |  |

**Table S2. Stress Activation in Female Participants with CAD (N = 53) During Mental Stress Compared to Control Tasks.**

|  |  |  | Talairach | | |  |
| --- | --- | --- | --- | --- | --- | --- |
| Voxel Number | **Brain Region** | **Brodmann Area** | **X** | **Y** | **Z** | **Z Score** |
| 963 | R Parietal Lobe, Postcentral Gyrus | 40 | 51 | -32 | 50 | Inf |
|  | R Parietal Lobe, Inferior Lobule | 40 | 48 | -56 | 45 | 7.51 |
|  | R Parietal Lobe, Inferior Lobule | 40 | 38 | -52 | 53 | 6.96 |
| 33 | L Cerebellum |  | -26 | -40 | -18 | Inf |
| 164 | L Cerebellum |  | -18 | -69 | -18 | 7.37 |
|  | L Cerebellum |  | -16 | -76 | -13 | 5.94 |
| 51 | R Cerebellum |  | 46 | -63 | -19 | 7.14 |
| 157 | R Frontal Lobe, Superior Frontal Gyrus | 9 | 42 | 37 | 32 | 7.04 |
|  | R Frontal Lobe, Middle Gyrus | 10 | 40 | 46 | 23 | 6.58 |
|  | R Frontal Lobe, Middle Gyrus | 9 | 44 | 29 | 34 | 6.02 |
| 218 | R Frontal Lobe, Inferior Gyrus | 44 | 59 | 12 | 13 | 6.78 |
|  | R Frontal Lobe, Inferior Gyrus | 45 | 59 | 20 | 9 | 6.26 |
|  | R Frontal Lobe, Inferior Gyrus | 45 | 51 | 22 | 12 | 6.12 |
| 164 | R Parietal Lobe, Precuneus | 7 | 4 | -51 | 38 | 6.69 |
|  | R Limbic Lobe, Cingulate Gyrus | 31 | 8 | -45 | 41 | 6.64 |
|  | R Parietal Lobe, Precuneus | 7 | 12 | -56 | 46 | 5.23 |
| 130 | R Parietal Lobe, Precuneus | 7 | 2 | -70 | 42 | 6.68 |
|  | L Parietal Lobe, Precuneus | 7 | -2 | -58 | 53 | 5.81 |
| 89 | L Frontal Lobe, Inferior Gyrus | 47 | -40 | 29 | -1 | 6.68 |
|  | L Frontal Lobe, Inferior Gyrus | 45 | -38 | 26 | 5 | 5.50 |
|  | L Frontal Lobe, Inferior Gyrus | 47 | -38 | 19 | -9 | 5.16 |
| 38 | R Cerebellum |  | 10 | -72 | -10 | 6.67 |
| 231 | R Parietal Lobe, Postcentral Gyrus | 3 | 53 | -9 | 49 | 6.61 |
|  | R Frontal Lobe, Middle Gyrus | 6 | 34 | -1 | 59 | 6.43 |
|  | R Frontal Lobe, Inferior Gyrus | 9 | 53 | 4 | 32 | 6.16 |
| 23 | L Cerebellum |  | -22 | -83 | -33 | 6.52 |
| 54 | L Frontal Lobe, Superior Gyrus | 10 | -22 | 63 | 12 | 6.49 |
| 25 | R Parietal Lobe, Postcentral Gyrus | 5 | 4 | -43 | 68 | 6.47 |
|  | L Parietal Lobe, Precuneus | 7 | -2 | -49 | 63 | 5.59 |
| 57 | R Frontal Lobe, Superior Gyrus | 8 | 42 | 20 | 46 | 6.42 |
| 31 | R Frontal Lobe, Precentral Gyrus | 6 | 59 | -10 | 42 | 6.34 |
| 70 | R Temporal Lobe, Superior Gyrus | 38 | 61 | 9 | -11 | 6.33 |
| 78 | L Frontal Lobe, Middle Gyrus | 11 | -24 | 32 | -12 | 6.27 |
|  | L Frontal Lobe, Inferior Gyrus | 11 | -20 | 30 | -18 | 5.47 |
|  | L Frontal Lobe, Inferior Gyrus | 47 | -28 | 25 | -6 | 5.21 |
| 21 | R Lentiform Nucleus |  | 24 | 6 | 11 | 6.27 |
| 24 | L Limbic Lobe, Anterior Cingulate | 32 | 0 | 41 | -2 | 6.27 |
| 22 | R Temporal Lobe, Superior Gyrus | 22 | 51 | -50 | 7 | 6.26 |
| 66 | R Limbic Lobe, Cingulate Gyrus | 32 | 6 | 14 | 40 | 6.25 |
| 19 | L Cerebellum |  | -38 | -77 | -18 | 6.19 |
| 186 | L Cerebellum |  | -46 | -46 | -31 | 6.19 |
|  | L Cerebellum |  | -46 | -54 | -26 | 5.84 |
|  | L Cerebellum |  | -53 | -44 | -33 | 5.57 |
| 27 | R Cerebellum |  | 4 | -53 | -2 | 6.12 |
|  | L Cerebellum |  | -6 | -61 | -7 | 5.70 |
| 14 | L Cerebellum |  | -44 | -62 | -34 | 6.12 |
| 18 | L Frontal Lobe, Precentral Gyrus | 6 | -36 | -12 | 41 | 6.08 |
| 13 | R Frontal Lobe, Superior Gyrus | 6 | 14 | 18 | 58 | 6.06 |
| 56 | L Temporal Lobe, Superior Gyrus | 38 | -34 | 24 | -28 | 6.04 |
| 13 | R Frontal Lobe, Middle Gyrus | 6 | 28 | 14 | 56 | 6.02 |
| 102 | R Frontal Lobe, Superior Gyrus | 8 | 14 | 48 | 36 | 6.01 |
|  | R Frontal Lobe, Superior Gyrus | 9 | 12 | 54 | 29 | 5.65 |
|  | R Frontal Lobe, Superior Gyrus | 9 | 20 | 54 | 33 | 5.51 |
| 36 | R Cerebellum |  | 22 | -75 | -15 | 6.00 |
| 20 | R Parietal Lobe, Inferior Lobule | 40 | 38 | -31 | 38 | 6.00 |
| 15 | R Temporal Lobe, Middle Gyrus | 39 | 40 | -65 | 15 | 5.98 |
| 31 | R Cerebellum |  | 24 | -50 | -23 | 5.97 |
| 15 | L Parietal Lobe, Supramarginal Gyrus | 40 | -55 | -51 | 33 | 5.93 |
| 61 | L Temporal Lobe, Fusiform Gyrus | 37 | -50 | -61 | -17 | 5.93 |
|  | L Cerebellum |  | -40 | -63 | -17 | 5.87 |
| 18 | L Temporal Lobe, Middle Gyrus | 20 | -55 | -35 | -8 | 5.87 |
| 11 | R Occipital Lobe, Middle Gyrus | 19 | 51 | -61 | 13 | 5.85 |
| 44 | L Frontal Lobe, Middle Gyrus | 46 | -46 | 23 | 26 | 5.85 |
|  | L Frontal Lobe, Inferior Gyrus | 45 | -46 | 20 | 17 | 5.28 |
| 14 | R Temporal Lobe, Superior Gyrus | 38 | 55 | 16 | -21 | 5.84 |
| 61 | R Frontal Lobe, Middle Gyrus | 47 | 53 | 46 | -6 | 5.81 |
|  | R Frontal Lobe, Inferior Gyrus | 46 | 50 | 43 | 10 | 5.79 |
|  | R Frontal Lobe, Inferior Gyrus | 46 | 55 | 45 | 3 | 5.11 |
| 29 | L Parietal Lobe, Postcentral Gyrus | 40 | -53 | -29 | 48 | 5.79 |
| 14 | L Parietal Lobe, Inferior Lobule | 40 | -61 | -33 | 33 | 5.79 |
| 74 | L Frontal Lobe, Middle Gyrus | 10 | -34 | 44 | 16 | 5.78 |
|  | L Frontal Lobe, Superior Gyrus | 10 | -34 | 55 | 12 | 5.59 |
| 102 | R Claustrum |  | 28 | 16 | 1 | 5.77 |
|  | R Insula |  | 42 | 18 | 1 | 5.54 |
|  | R Claustrum |  | 34 | 14 | 6 | 5.34 |
| 15 | R Temporal Lobe, Sub-Gyral | 37 | 48 | -45 | -10 | 5.77 |
| 26 | L Temporal Lobe, Superior Gyrus | 39 | -44 | -57 | 28 | 5.73 |
| 37 | R Frontal Lobe, Middle Gyrus | 9 | 51 | 17 | 26 | 5.71 |
| 16 | R Frontal Lobe, Superior Gyrus | 10 | 8 | 61 | 24 | 5.70 |
| 21 | R Frontal Lobe, Superior Gyrus | 10 | 20 | 65 | 12 | 5.69 |
| 14 | R Limbic Lobe, Uncus | 38 | 22 | 2 | -37 | 5.68 |
| 66 | R Temporal Lobe, Middle Gyrus | 22 | 55 | -33 | 2 | 5.65 |
|  | R Temporal Lobe, Middle Gyrus | 22 | 51 | -39 | -1 | 5.56 |
|  | R Temporal Lobe, Middle Gyrus | 22 | 65 | -29 | 5 | 5.54 |
| 38 | R Parietal Lobe, Precuneus | 7 | 24 | -56 | 46 | 5.60 |
|  | R Parietal Lobe, Superior Lobule | 7 | 28 | -54 | 40 | 5.19 |
| 23 | R Occipital Lobe, Cuneus | 7 | 16 | -72 | 30 | 5.57 |
| 20 | L Frontal Lobe, Superior Gyrus | 9 | -12 | 58 | 27 | 5.57 |
| 15 | R Frontal Lobe, Superior Gyrus | 10 | 28 | 56 | 22 | 5.53 |
| 12 | R Frontal Lobe, Middle Gyrus | 47 | 48 | 37 | -5 | 5.48 |
| 12 | L Temporal Lobe, Fusiform Gyrus | 37 | -44 | -65 | -9 | 5.47 |
| 11 | R Cerebellum |  | 14 | -32 | -19 | 5.47 |
| 18 | L Frontal Lobe, Precentral Gyrus | 6 | -40 | 0 | 30 | 5.46 |
| 12 | L Occipital Lobe, Middle Gyrus | 19 | -48 | -76 | -3 | 5.40 |
| 16 | R Frontal Lobe, Medial Gyrus | 6 | 14 | 8 | 48 | 5.37 |
| 21 | L Temporal Lobe, Superior Gyrus | 22 | -53 | -27 | 6 | 5.34 |
|  | L Temporal Lobe, Superior Gyrus | 22 | -48 | -17 | 4 | 5.32 |
| 15 | L Temporal Lobe, Middle Gyrus | 21 | -51 | -8 | -13 | 5.30 |
|  | L Temporal Lobe, Inferior Gyrus | 21 | -59 | -12 | -16 | 5.15 |
| 12 | L Cerebellum |  | -18 | -51 | -16 | 5.25 |

| *Number of voxels in the highlighted cluster. Only clusters with a minimum of 11 contiguous voxels were considered an activation. | |
| --- | --- |
| ** one tailed Z-score > 5.7, p < 0.005 with family-wise error rate correction |  |

**Table S3. Stress Deactivation in Female Participants with CAD (N = 53) During Mental Stress Compared to Control Tasks.**

|  |  | | |  | | **Talairach** | | | | | |  | | |
| --- | --- | --- | --- | --- | --- | --- | --- | --- | --- | --- | --- | --- | --- | --- |
| **Voxel Number*** | **Brain Region** | | | **Brodmann’s Area** | | **X** | **Y** | | **Z** | | | | **Z score**** | |
| 133 | R Frontal Lobe, Inferior Gyrus | | | 47 | | 24 | 22 | | -20 | | | | 7.27 | |
|  | R Frontal Lobe, Orbital Gyrus | | | 47 | | 22 | 30 | | -23 | | | | 6.17 | |
| 88 | L Frontal Lobe, Medial Gyrus | | | 6 | | -14 | 29 | | 36 | | | | 7.17 | |
|  | L Frontal Lobe, Superior Gyrus | | | 8 | | -12 | 32 | | 47 | | | | 5.90 | |
|  | L Limbic Lobe, Cingulate Gyrus | | | 32 | | -10 | 22 | | 44 | | | | 5.15 | |
| 52 | R Frontal Lobe, Superior Gyrus | | | 6 | | 14 | 22 | | 51 | | | | 7.12 | |
| 335 | L Parietal Lobe, Sub-Gyral | | | 40 | | -26 | -38 | | 49 | | | | 7.10 | |
|  | L Parietal Lobe, Precuneus | | | 7 | | -14 | -44 | | 59 | | | | 6.97 | |
|  | L Parietal Lobe, Precuneus | | | 7 | | -10 | -40 | | 47 | | | | 6.66 | |
| 49 | L Parietal Lobe, Supramarginal Gyrus | | | 40 | | -63 | -43 | | 27 | | | | 7.06 | |
|  | L Temporal Lobe, Supramarginal Gyrus | | | 40 | | -63 | -49 | | 23 | | | | 6.33 | |
| 159 | R Temporal Lobe, Superior Gyrus | | | 42 | | 63 | -28 | | 16 | | | | 7.02 | |
|  | R Insula | | | 13 | | 53 | -34 | | 17 | | | | 5.35 | |
| 347 | R Frontal Lobe, Precentral Gyrus | | | 43 | | 57 | -5 | | 10 | | | | 6.85 | |
|  | R Temporal Lobe, Superior Gyrus | | | 22 | | 65 | -1 | | 10 | | | | 6.29 | |
|  | R Parietal Lobe, Postcentral Gyrus | | | 43 | | 59 | -5 | | 17 | | | | 6.06 | |
| 189 | L Frontal Lobe, Orbital Gyrus | | | 11 | | -6 | 44 | | -19 | | | | 6.84 | |
|  | L Frontal Lobe, Medial Gyrus | | | 11 | | -4 | 52 | | -14 | | | | 6.16 | |
|  | R Frontal Lobe, Medial Gyrus | | | 10 | | 4 | 52 | | -9 | | | | 6.02 | |
| 28 | L Anterior Cingulate | | | 24 | | -4 | 23 | | 24 | | | | 6.79 | |
| 91 | R Frontal Lobe, Inferior Gyrus | | | 47 | | 44 | 27 | | -6 | | | | 6.77 | |
|  | R Frontal Lobe, Inferior Gyrus | | | 47 | | 50 | 28 | | -15 | | | | 6.33 | |
| 148 | L Thalamus | | |  | | -16 | -27 | | 3 | | | | 6.76 | |
|  | L Thalamus | | |  | | -8 | -21 | | 4 | | | | 6.08 | |
|  | L Thalamus | | |  | | -10 | -29 | | 10 | | | | 5.14 | |
| 152 | R Frontal Lobe, Subcallosal Gyrus | | | 34 | | 18 | 7 | | -14 | | | | 6.67 | |
|  | R Anterior Cingulate | | | 25 | | 4 | 11 | | -9 | | | | 6.20 | |
|  | R Caudate | | |  | | 8 | 6 | | -4 | | | | 5.16 | |
| 55 | L Frontal Lobe, Superior Gyrus | | | 6 | | -6 | 12 | | 55 | | | | 6.62 | |
| 55 | R Temporal Lobe, Middle Gyrus | | | 21 | | 69 | -45 | | -10 | | | | 6.62 | |
|  | R Temporal Lobe, Middle Gyrus | | | 21 | | 71 | -43 | | -3 | | | | 5.53 | |
| 35 | R Frontal Lobe, Medial Gyrus | | | 9 | | 22 | 38 | | 23 | | | | 6.54 | |
| 17 | L Parietal Lobe, Superior Lobule | | | 7 | | -26 | -64 | | 50 | | | | 6.53 | |
| 117 | R Frontal Lobe, Middle Gyrus | | | 11 | | 42 | 46 | | -17 | | | | 6.50 | |
|  | R Frontal Lobe, Middle Gyrus | | | 10 | | 44 | 50 | | -7 | | | | 5.73 | |
|  | R Frontal Lobe, Middle Gyrus | | | 11 | | 36 | 58 | | -15 | | | | 5.70 | |
| 53 | R Frontal Lobe, Paracentral Lobule | | | 5 | | 16 | -39 | | 46 | | | | 6.43 | |
|  | R Frontal Lobe, Paracentral Lobule | | | 5 | | 8 | -32 | | 52 | | | | 6.19 | |
| 37 | L Temporal Lobe, Fusiform Gyrus | | | 37 | | -38 | -57 | | -11 | | | | 6.38 | |
| 76 | R Temporal Lobe, Inferior Gyrus | | | 21 | | 59 | -7 | | -16 | | | | 6.37 | |
| 58 | L Frontal Lobe, Inferior Gyrus | | | 47 | | -51 | 18 | | 1 | | | | 6.35 | |
| 40 | L Frontal Lobe, Medial Gyrus | | | 10 | | -4 | 55 | | 6 | | | | 6.35 | |
| 91 | R Lentiform Nucleus | | |  | | 32 | -13 | | 2 | | | | 6.31 | |
|  | R Insula | | | 13 | | 42 | -17 | | 3 | | | | 5.50 | |
| 120 | L Insula | | | 13 | | -32 | -21 | | 14 | | | | 6.30 | |
|  | L Temporal Lobe, Superior Gyrus | | | 41 | | -36 | -34 | | 15 | | | | 5.66 | |
|  | L Temporal Lobe, Superior Gyrus | | | 13 | | -38 | -23 | | 8 | | | | 5.48 | |
| 147 | L Parahippocampal Gyrus | | | 30 | | -12 | -48 | | 6 | | | | 6.29 | |
|  | L Cerebellum | | |  | | -4 | -41 | | 2 | | | | 5.55 | |
|  | L Posterior Cingulate | | | 29 | | -4 | -38 | | 10 | | | | 5.46 | |
| 144 | R Posterior Cingulate | | | 29 | | 8 | -48 | | 11 | | | | 5.53 | |
|  | R Parahippocampal Gyrus | | | 30 | | 12 | -44 | | 5 | | | | 5.21 | |
| 52 | R Thalamus | | |  | | 14 | -7 | | 5 | | | | 6.23 | |
|  | R Thalamus | | |  | | 6 | -8 | | -1 | | | | 5.14 | |
| 57 | R Occipital Lobe, Middle Gyrus | | | 19 | | 50 | -68 | | 8 | | | | 6.18 | |
| 65 | L Frontal Lobe, Precentral Gyrus | | | 4 | | -44 | -16 | | 39 | | | | 6.13 | |
| 77 | L Frontal Lobe, Inferior Gyrus | | | 44 | | -61 | 14 | | 9 | | | | 6.11 | |
|  | L Frontal Lobe, Precentral Gyrus | | | 6 | | -59 | 0 | | 7 | | | | 5.66 | |
| 259 | L Limbic Lobe, Posterior Cingulate | | | 30 | | -14 | -54 | | 14 | | | | 6.11 | |
|  | R Occipital Lobe, Cuneus | | | 18 | | 6 | -77 | | 19 | | | | 5.92 | |
|  | L Parietal Lobe, Precuneus | | | 31 | | -4 | -61 | | 23 | | | | 5.90 | |
| 13 | R Parietal Lobe, Inferior Lobule | | | 40 | | 32 | -38 | | 49 | | | | 6.07 | |
| 25 | R Cerebellum | | |  | | 26 | -70 | | -39 | | | | 6.06 | |
|  | R Cerebellum | | |  | | 22 | -73 | | -32 | | | | 5.22 | |
| 37 | L Occipital Lobe, Lingual Gyrus | | | 18 | | -4 | -81 | | 4 | | | | 6.04 | |
|  | L Occipital Lobe, Lingual Gyrus | | | 17 | | -10 | -87 | | 4 | | | | 5.22 | |
| 37 | R Temporal Lobe, Superior Gyrus | | | 22 | | 51 | 12 | | -2 | | | | 6.01 | |
| 27 | R Frontal Lobe, Precentral Gyrus | | | 4 | | 40 | -14 | | 36 | | | | 5.99 | |
| 138 | R Insula | | | 13 | | 40 | 2 | | 0 | | | | 5.97 | |
| *Number of voxels in the highlighted cluster. Only clusters with a minimum of 11 contiguous voxels were considered an activation. | | | | | | | | | | | | | |  |
| ** one tailed Z-score > 5.7, p < 0.005 with family-wise error rate correction | |  |  |  |  |  |  |  |  |  |  |  |  |  |
| **Table S4. Stress Activation in Male Participants with CAD (N = 112) During Mental Stress Compared to Control Tasks.** | | | | | | | | | | | | | |  |
|  |  | |  | |  | | | **Talairach** | |  |  | | | |
| **Voxel Number*** | **Brain Regions** | | **Brodmann’s Area** | | **X** | | | **Y** | | **Z** | **Z Score**** | | | |
| 93780 | L Anterior Cingulate Gyrus | | 32 | | -4 | | | 18 | | 39 | 65535 | | | |
|  | L Frontal Lobe, Cingulate Gyrus | | 32 | | 0 | | | 25 | | 36 | 65535 | | | |
|  | R Frontal Lobe, Middle Gyrus | | 10 | | 34 | | | 53 | | 6 | 65535 | | | |
| 92 | L Temporal Lobe, Superior Gyrus | | 41 | | -38 | | | -36 | | 15 | 6.95 | | | |
|  | L Temporal Lobe, Transverse Gyrus | | 41 | | -34 | | | -29 | | 6 | 5.77 | | | |
|  | L Temporal Lobe, Superior Gyrus | | 13 | | -44 | | | -42 | | 17 | 5.68 | | | |
| 104 | R Occipital Lobe, Lingual Gyrus | | 18 | | 4 | | | -72 | | 5 | 6.67 | | | |
|  | R Occipital Lobe, Cuneus | | 30 | | 8 | | | -62 | | 6 | 6.03 | | | |
|  | R Cerebellum | |  | | 6 | | | -62 | | -2 | 5.83 | | | |
| 29 | L Thalamus | |  | | -18 | | | -11 | | 17 | 6.44 | | | |
| 46 | L Cerebellum | |  | | -2 | | | -48 | | 4 | 6.40 | | | |
| 25 | R Cerebellum | |  | | 53 | | | -62 | | -39 | 6.06 | | | |
|  | R Cerebellum | |  | | 53 | | | -68 | | -32 | 5.93 | | | |
| 28 | R Cerebellum | |  | | 38 | | | -37 | | -35 | 6.06 | | | |
| 45 | R Cerebellum | |  | | 55 | | | -71 | | -25 | 5.94 | | | |
|  | R Cerebellum | |  | | 50 | | | -73 | | -20 | 5.93 | | | |
| 20 | L Cerebellum | |  | | -32 | | | -39 | | -40 | 5.92 | | | |
| 29 | L Occipital Lobe, Lingual Gyrus | | 18 | | -2 | | | -80 | | -3 | 5.86 | | | |
| 29 | R Limbic Lobe, Parahippocampal Gyrus | | 30 | | 10 | | | -41 | | 4 | 5.72 | | | |
|  | R Thalamus | |  | |  | | |  | |  |  | | | |
|  | | | | | | | | | | | | | |  |
| *Number of voxels in the highlighted cluster. Only clusters with a minimum of 11 contiguous voxels were considered an activation. | | | | | | | | | | | | | |  |
| **one tailed Z-score > 5.7, p < 0.005 with family-wise error rate correction | |  |  |  |  |  |  |  |  |  |  |  |  |  |

| **Table S5. Stress Deactivation in Male Participants with CAD (N = 112) During Mental Stress Compared to Control Tasks.** | | | | | | | | | |
| --- | --- | --- | --- | --- | --- | --- | --- | --- | --- |
|  | | |  | **Talairach** | | | |  | |
| **Voxel Number*** | **Brain Regions** | | **Brodmann’s Area** | **X** | **Y** | **Z** | **Z score**** | | |
| 96282 | L Occipital Lobe, Lingual Gyrus | | 18 | -22 | -82 | -1 | 65535 | | |
|  | R Occipital Lobe, Inferior Gyrus | | 18 | 32 | -78 | -5 | 65535 | | |
|  | L Occipital Lobe, Lingual Gyrus | | 18 | -30 | -74 | -5 | 65535 | | |
| 151 | L Frontal Lobe, Middle Gyrus | | 8 | -46 | 21 | 41 | 7.23 | | |
|  | L Frontal Lobe, Middle Gyrus | | 46 | -51 | 25 | 26 | 6.83 | | |
|  | L Frontal Lobe, Superior Gyrus | | 9 | -42 | 38 | 32 | 6.52 | | |
| 292 | R Frontal Lobe, Superior Gyrus | | 10 | 10 | 70 | -3 | 7.02 | | |
|  | R Frontal Lobe, Superior Gyrus | | 10 | 6 | 70 | 7 | 7.00 | | |
|  | R Frontal Lobe, Superior Gyrus | | 10 | 24 | 59 | 7 | 6.72 | | |
| 26 | L Frontal Lobe, Precentral Gyrus | | 4 | -18 | -23 | 50 | 6.97 | | |
| 20 | L Frontal Lobe, Medial Gyrus | | 10 | -14 | 53 | 13 | 6.81 | | |
| 63 | L Frontal Lobe, Superior Gyrus | | 8 | -32 | 26 | 49 | 6.79 | | |
|  | L Frontal Lobe, Superior Gyrus | | 8 | -34 | 18 | 51 | 6.47 | | |
| 27 | L Frontal Lobe, Precentral Gyrus | | 6 | -30 | 9 | 26 | 6.35 | | |
| 87 | R Frontal Lobe, Superior Gyrus | | 9 | 22 | 38 | 32 | 6.30 | | |
|  | R Frontal Lobe, Superior Gyrus | | 8 | 28 | 45 | 38 | 5.92 | | |
|  | R Frontal Lobe, Middle Gyrus | | 8 | 34 | 39 | 38 | 5.56 | | |
| 21 | R Frontal Lobe, Precentral Gyrus | | 6 | 40 | 3 | 25 | 6.30 | | |
| 30 | R Parietal Lobe, Inferior Lobule | | 39 | 50 | -62 | 38 | 6.29 | | |
| 34 | L Frontal Lobe, Superior Gyrus | | 10 | -28 | 64 | 0 | 6.28 | | |
| 47 | L Frontal Lobe, Superior Gyrus | | 8 | -18 | 45 | 40 | 6.20 | | |
|  | L Frontal Lobe, Superior Gyrus | | 8 | -24 | 35 | 45 | 6.01 | | |
| *Number of voxels in the highlighted cluster. Only clusters with a minimum of 11 contiguous voxels were considered an activation. | | | | | | | | |  |
| ** one tailed Z-score > 5.7, p < 0.005 with family-wise error rate correction | |  |  |  |  |  |  |  |  |
